# Orbital cavernous hemangioma in an infant with intracranial lesions: a case report

**DOI:** 10.4076/1757-1626-2-6912

**Published:** 2009-09-11

**Authors:** Eleni Evagelidou, Elena Tsanou, Ioannis Asproudis, Spiridon Gorezis, Miltiadis Aspiotis, Dimitrios Peschos, Antigoni Siamopoulou

**Affiliations:** 1 Pediatric Clinic, University Hospital of Ioannina, Panepistimiou Avenue, Ioannina, 45 500, Greece; 2 Cytology-Pathology Department, University Hospital of Ioannina, Panepistimiou Avenue, Ioannina, 45 500, Greece; 3 Eye Clinic, University Hospital of Ioannina, Panepistimiou Avenue, Ioannina, 45 500, Greece; 4 Epitrus Vision Center, Panepistimiou Avenue, Ioannina, 45 500, Greece

## Abstract

**Introduction:**

Cavernous hemangiomas of the orbit are benign vascular malformations, commonly encountered in adults. Although they are infrequent in pediatric population their diagnosis and course are of a great significance, mainly because they can cause visual disturbances such as amblyopia that can ensue, and secondarily due to their cosmetic and psychological effect. Special attention is required in follow up and treatment. Additionally, a systemic evaluation is necessary in order to discover asymptomatic lesions elsewhere in the body carrying a risk of complications.

**Case presentation:**

The authors describe the clinical course, diagnosis, therapeutic approach and prognosis of an infant with an orbital cavernous hemangioma accompanying intracranial lesions. A female infant 18 months of age, presented with a mass in the left upper eyelid, causing blepharoptosis. Preoperative magnetic resonance imaging and angiography of the brain and the orbits showed a hemangioma of the left upper eyelid and intracranial lesions to the left temporal fossa and the pons. At the age of 2 years and 8 months she was admitted again due to severe eyelid swelling, intense strong pain, exophthalmos and collateral ophthalmoplegia. Two operations were performed to remove the orbit mass. Histological examination, showed characteristics of cavernous hemangioma.

**Conclusion:**

The atypical presentation of cavernous orbital hemangioma with early infantile onset, merits attention.

## Introduction

Cavernous hemangiomas are uncommon vascular malformations located in the brain [1]. They are usually encountered in the orbit as primary tumours in adults [1,2]. Patients with orbital cavernous hemangiomas typically present in the fourth and fifth decade of life [1,2]. Lesions occurring under the age of 20 – and especially in infants are rare [1]-[4].

We describe a case of a female infant with an orbital cavernous hemangioma and accompanying intracranial lesions, which was implicated with serious thrombosis, optic nerve atrophy, severe visual loss and strabismus.

## Case presentation

A Greek female infant, 18 months of age, was referred to the pediatric clinic for evaluation of a mass in the left upper eyelid.

According to the patient's perinatal and medical history, she was born after a 36-weeks twin pregnancy, following in-vitro fertilization (IVF), with a birth weight of 2.200 g, small-for-gestational age. There was no positive family history of hemangiomas. There were no indications of deficiency in her developmental status. Also there was no history of trauma, seizures or focal neurological deficits.

The physical examination revealed no pathologic findings with the exception of a soft and painless mass in the left upper eyelid, causing incomplete closure of the eyelid and only mild irregular discoloration of the lid skin. An increase in tumor volume and depth of bluish hue when the child cried were noticeable. Also there was a small subcutaneous mass in the upper lip and a café-au-lait spot with smooth margins on the right leg. No other skin spots were observed.

The ophthalmological evaluation showed that both eyes were equal in size and eye motility was normal. No strabismus was recognized. Direct ophthalmoscopy showed no abnormality in either anterior segment. The fundus examination in both eyes was normal. Skiascopic examination revealed no particular refractive errors, except for a small degree of hyperopia.

**Figure 1:**
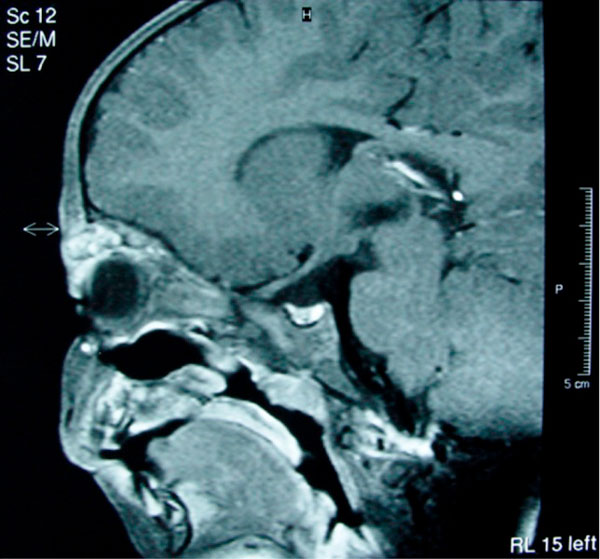
**Cavernous hemangioma of the left upper eyelid on a sagittal T1 –weighted MRI**.

**Figure 2:**
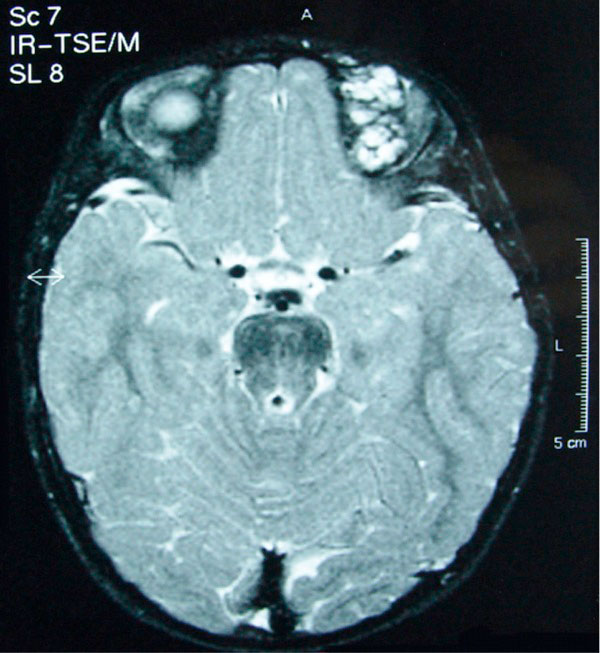
**Cavernous hemangioma of the left upper eyelid in the same patient, on an axial T2- weighted MRI, after intravenous injection of Gadolinium**.

Subsequent examination of the skin and magnetic resonance imaging (MRI) of the brain and the orbits (with T1 and T2 weighted sequences) and magnetic resonance angiography (MRA) were performed. A lesion resembling cavernous hemangioma was found in the superior part of the left orbit (Figure 1). The lesion was located between the roof of the orbit and the superior rectus muscle. The lesion was dyed with gadolinium approximately 13 min after infusion of the agent (Figure 2). No dilatation of the collateral ophthalmic vein was observed. A similar lesion was found intracranially located in the left temporal fossa, extending to the collateral cavernous sinus up to the lateral wall of the fourth ventricle through the cerebellopontine angle. This finding resembled a cavernous angioma. No pathologic findings were detected in cerebellar and cerebral hemispheres. The lesion was also extending to the pons. A similar lesion was found in the upper lip. No signs of hemangioma or other pathologic findings were noted in the abdomen, pelvis and post-peritoneum by ultrasonography. Furthermore there was no evidence for presence of other diseases such as type-1 neurofibromatosis, Sturge-Weber syndrome, Blue rubber bleb nevus syndrome or von Hippel-Lindau syndrome.

By 23 months of age, our patient required hospitalization due to increasing size of the orbital hemangioma causing severe reduction of the palpebral fissure. A second MRI of the brain and orbits revealed increasing in size of the hemangioma in its lateral palpebral part, especially in the part localized outside the orbit. There were no alterations in the intracranial lesions.

**Figure 3:**
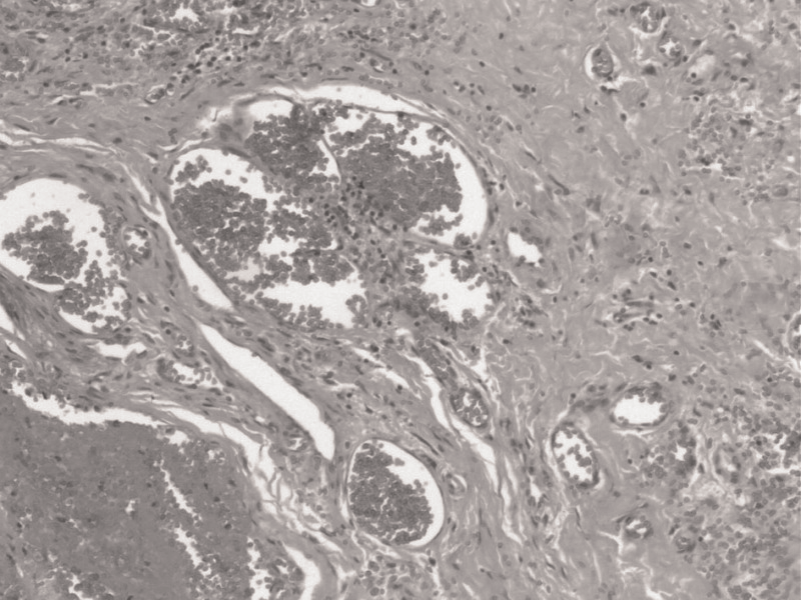
**Large vascular spaces lined by endothelial cells**. Note the red blood cells at the periphery of the lumen.

At the age of 2 years and 8 months, our patient was admitted again due to sudden and severe left upper eyelid swelling, intense pain and exophthalmos in the left eye. Clinical examination revealed alterations of ocular motility and pupil dilation, while imaging suggested the presence of intraorbital hemorrhage. The patient was rushed to surgery on order to decompensate the orbit and protect the optic nerve. An anterior surgical approach was performed through the superior orbital rim allowing entrance to the roof of the orbit and removal of a dark red mass. On histological examination, the lesion proved to be a cavernous hemangioma (Figure 3). Large anastomosing vascular spaces were observed filled with blood and separated by fibrous stroma.

The excision proved to be incomplete and the postoperative course was not uneventful. The patient presented with consistent eyelid swelling, exophthalmos and collateral ophthalmoplegia. A second operation was done in order to remove the remnants of the cavernous hemangioma, twenty days postoperatively. Two years after the operations, the patient shows mild palpebral ptosis, visual acuity of 1/20- 1/10 (left eye) and 10/10 (right eye) in the Snellen optotype, total atrophy of the left optic nerve and small angle divergent strabismus.

## Discussion

Cavernous hemangiomas are non-infiltrative, low-flow hamartomas, usually observed in adults and thought to be benign [5]. In the orbit, they represent 80% of adult angiomatous malformations, accounting for 9.5% to 15% of primary orbital expanding masses [1,5]. Orbital cavernomas have been reported to appear with symptoms at an average age of 42 years, ranging from 18 to 67 years [6]. Intracranial and orbital cavernous hemangiomas in children, especially in infants, are rare [4]. The prevalence of cerebral cavernous malformations in children is estimated to be between 0.37% and 0.53% [7]. Multiple lesions in a single orbital cavity or simultaneously occurring in both orbits have been reported, while a familial form of orbital cavernous angiomas have also been described [6,8]. The incidence of familial cerebral cases has been estimated to be close to 20% in the literature, and the pattern of inheritance is consistent with an autosomal dominant mode with incomplete clinical penetration and possible de novo mutation [7].

In our patient there was a combination of early age of onset (infancy), cavernous hemangiomas in orbit and lip, cerebral cavernoma with lesions in left temporal fossa, left cavernous sinus and pons, and asymptomatic clinical profile in relation to cerebral angiomas. The examinations for hemangiomas elsewhere in the body and the familial history of clinical noticeable hemangiomas were negative.

The pathogenesis of orbital cavernous angiomas is uncertain [1]. Gardner has postulated that cavernous hemangiomas grow slowly possibly from a pre-existing vascular hamartomas by intracapillary endothelial hyperplasia and they are relatively isolated from the systemic circulation [9]. Harris and Jackobiec suggested that these angiomas are acquired lesions that probably begin as capillary-type proliferation, becoming cavernous spaces through a progressive ectasia [1,5].

The natural history of orbital cavernous hemangiomas may be quite variable. It may be that of benign tumours causing progressive clinical manifestations from mass effect due to their slow growth or sometimes they may not grow after a certain age or remain asymptomatic [2,5,11]. The reported stimulation in their growth during pregnancy seems to suggest some hormonal influence on their natural evolution [12].

Children with an infantile hemangioma in the eyelid and orbit are at risk of a variety of ocular problems including functional amblyopia, strabismus, proptosis and optic nerve compression [13,14]. The mal-positioned lids may lead to the development of amblyopia from partial or complete occlusion of the visual axis. The tumor may also interfere with normal visual function by leading to astigmatism from pressing on the eye and altering the curvature of the cornea. Myopia and strabismus may secondarily relate to these hemangiomas as well [1,13]-[14]. On occasion, symptoms of orbital pain, eyelid swelling, diplopia and amaurosis can occur. Patients may refer orbital pain or headache, and choroidal folds or retinal striae, as well as optic disc edema may also be seen [1][6]. Orbital cavernous hemangiomas have no tendency to cause hemorrhages, and acute clinical onset is rare [1]. In our case, the patient ended two years after the operation to show small angle divergent strabismus and atrophy of the left optic nerve. This atrophy may be the result of the operative procedure or result of the compression of the optic nerve or its vascular supply by the tumor.

The diagnosis and follow up imaging of cavernous angiomas (intracranial and orbital cavernous angiomas) are best provided by MRI [7]. In our pediatric patient, MRI scanning including T1-, T2-contrast-enchanced and T1-, T2-weighted sequences were done. Also MRA was performed in order to assess the anatomical rapport between cavernomas and the normal arterial vessels. Cavernous angiomas are well known to be angiographically silent, perhaps due either to the small caliber of the feeding arteries and their slow circulation causing dilution of contrast medium, to extensive thrombosis or to a combination of both [4].

Various methods have been used to treat infantile hemangiomas of the eyelid and orbit. According to Herter et al. [6], operative treatment is recommended particularly in patients with true visual loss secondary to optic nerve compression, severe bulbar displacement and constant growth tendency.

The choice of management in cases of cerebral cavernous angiomas must take into account both the possible natural evolution of the lesions and the risk of surgery. Therapeutic strategies for cerebral cavernomas take into account age, sex, location of the lesions, the efficacy of medical treatment for patient's secondary epilepsy and risk factors for severe potentially life-threatening hemorrhage [7]. In children, surgery is clearly indicated in case of acute hemorrhage or focal neurological deficit [7]. It is especially recommended for infratentorial lesions, even if they are clinically silent, due to their high risk of bleeding. However, the surgical indication must be always discussed for each case individually

## Conclusion

Cavernous hemangiomas of the orbit are benign vascular malformations, commonly encountered in adults. Although they are infrequent in pediatric population their diagnosis and course are of a great significance, mainly because they can cause visual disturbances such as amblyopia that can ensue, and secondarily due to their cosmetic and psychological effect. Special attention is required in follow up and treatment. Additionally, a systemic evaluation is necessary in order to discover asymptomatic lesions elsewhere in the body carrying a risk of complications.

## Abbreviations

IVF: in-vitro fertilization; MRA: magnetic resonance angiography; MRI: magnetic resonance imaging.

## Consent

Written informed consent was obtained from the patient for publication of this case report and accompanying images. A copy of the written consent is available for review by the editor in chief of this journal.

## Competing interests

The authors declare that they have no competing interests.

## Authors' contributions

"EE was a major contributor in writing the paper; ET performed the histologic examination; IA analysed and interpreted the visual data of the patient; SG contributed to final revision of the paper; MA provided the final approval for the version to be published, DP provided the histological images and AS concepted and designed the study".
